# Specific Alterations in Complement Protein Activity of Little Brown Myotis (*Myotis lucifugus*) Hibernating in White-Nose Syndrome Affected Sites

**DOI:** 10.1371/journal.pone.0027430

**Published:** 2011-11-30

**Authors:** Marianne S. Moore, Jonathan D. Reichard, Timothy D. Murtha, Bita Zahedi, Renee M. Fallier, Thomas H. Kunz

**Affiliations:** Department of Biology, Center for Ecology and Conservation Biology, Boston University, Boston, Massachusetts, United States of America; New York State Health Department and University at Albany, United States of America

## Abstract

White-nose syndrome (WNS) is the most devastating condition ever reported for hibernating bats, causing widespread mortality in the northeastern United States. The syndrome is characterized by cutaneous lesions caused by a recently identified psychrophilic and keratinophylic fungus (*Geomyces destructans*), depleted fat reserves, atypical behavior, and damage to wings; however, the proximate cause of mortality is still uncertain. To assess relative levels of immunocompetence in bats hibernating in WNS-affected sites compared with levels in unaffected bats, we describe blood plasma complement protein activity in hibernating little brown myotis (*Myotis lucifugus*) based on microbicidal competence assays using *Escherichia coli*, *Staphylococcus aureus* and *Candida albicans*. Blood plasma from bats collected during mid-hibernation at WNS-affected sites had higher bactericidal ability against *E. coli* and *S. aureus*, but lower fungicidal ability against *C. albicans* when compared with blood plasma from bats collected at unaffected sites. Within affected sites during mid-hibernation, we observed no difference in microbicidal ability between bats displaying obvious fungal infections compared to those without. Bactericidal ability against *E. coli* decreased significantly as hibernation progressed in bats collected from an affected site. Bactericidal ability against *E. coli* and fungicidal ability against *C. albicans* were positively correlated with body mass index (BMI) during late hibernation. We also compared complement activity against the three microbes within individuals and found that the ability of blood plasma from hibernating *M. lucifugus* to lyse microbial cells differed as follows: *E. coli*>*S. aureus*>*C. albicans*. Overall, bats affected by WNS experience both relatively elevated and reduced innate immune responses depending on the microbe tested, although the cause of observed immunological changes remains unknown. Additionally, considerable trade-offs may exist between energy conservation and immunological responses. Relationships between immune activity and torpor, including associated energy expenditure, are likely critical components in the development of WNS.

## Introduction

White-nose syndrome (WNS) is the most devastating disease ever reported for wildlife in North America—with mortality of hibernating bats approaching 100% at some hibernacula in the northeastern United States [1], [2], [3]. This emergent infectious disease is associated with a newly described psychrophilic and keratinophylic fungus, *Geomyces destructans* 4,5. Bats infected with *G. destructans* were first recorded photographically in February 2006 from Howes Cave located approximately 52 km west of Albany, New York [1]. As of March 2011, presence of *G. destructans* in North America has been confirmed in 16 states (Connecticut, Delaware, Indiana, Maryland, Massachusetts, Missouri, New Hampshire, New Jersey, New York, North Carolina, Oklahoma, Pennsylvania, Tennessee, Vermont, Virginia, and West Virginia), three Canadian provinces (New Brunswick, Ontario, Quebec) and from nine hibernating bat species. WNS is manifested by some or all of the following symptoms: 1) a cutaneous fungal infection appearing as white, filamentous hyphae with distinct conidia (spores) on the nose, ears, and wing membranes [3], [4], [5], [6]; 2) depleted white and brown fat reserves [3], [7], [8] (Jonathan D. Reichard, unpublished data); 3) ulcerated, necrotic and scarred wing membranes [9]; and 4) atypical behavior causing bats to emerge prematurely from hibernacula in mid-winter (Alan C. Hicks, unpublished data). *G. destructans* grows on the skin (nose, ears, and wing membranes) of hibernating bats, and laboratory research has revealed that it grows optimally at temperatures characteristic of hibernacula, ranging from 2 to 14°C [3]. Histopathological evidence, the gold standard of infectivity [8], indicates that the fungus sometimes penetrates the dermis and underlying connective tissue of hibernating bats especially in areas associated with sebaceous glands and hair follicles, but does not appear to elicit an inflammatory immune response. Recently, *G. destructans* was isolated from six bat species in Europe [10], [11], [12]; however, except for the presence of cutaneous fungal growth, other symptoms associated with WNS in North America have not been reported in Europe. While understanding of the causes and consequences of WNS is incomplete, over one million bats have died from this disease with serious ecological and economic consequences expected, including the predicted extinction of at least one species within two decades [2].

Although a physiological disruption, changes in environmental conditions, or a combination of factors may be involved in the development of the syndrome, recent evidence supports that WNS is caused by the infectious fungal pathogen *G. destructans* [5], [13]. Thus, it is important to understand levels of immunocompetence in bats affected with WNS and to describe their potential for resisting pathogenic or opportunistic fungal infections. Studies conducted primarily in humans and mice demonstrate that immunological defense against invading fungi begins, in part, with the activation of soluble complement proteins resulting in membrane lysis of foreign cells, increased cell signaling and adhesion, designation of pathogens for phagocytic killing, a decreased threshold for B cell activation, an effective proinflammatory response, and the clearance of immune complexes and cell debris [14], [15], [16], [17]. In addition to complement protein activity, successful immune response against invading fungi includes inflammatory mechanisms [18], direct antifungal effector functions provided by phagocytic cells (e.g. macrophages, neutrophils, dendritic cells) [19], T lymphocyte cell-mediated cytotoxicity [16], [17], [18], [20], and antibody-dependent cellular cytotoxicity [16], [17].

As seasonal hibernators, North American bat species currently affected by WNS (*Myotis lucifugus* (Le Conte), *M. septentrionalis* (Trouessart), *M. sodalis* (Miller and Allen), *M. leibii* (Audubon and Bachman), *Perimyotis subflavus* (Cuvier), and *Eptesicus fuscus* (de Beauvois) [1], [3], [4] may experience levels of immunocompetence constrained by prolonged periods of deep torpor. Although no published studies have addressed the possible effects of hibernation specifically on the immune system of free-ranging bats, research in captive bats [21] and in other hibernating mammals suggest that deep torpor affects numerous mechanisms associated with immunity [22], [23], [24], [25], [26], [27] and experimental studies show that immunological challenge causes alterations in the length of interbout arousals [28], [29]. Physiological adjustments associated with deep torpor can result in a decreased primary [30] and secondary [28], [31] humoral response, an absence of T and B lymphocyte proliferative ability [26], and a decrease in serum complement protein activity [24]. Torpor can also result in increased rates of infection [32], [33], [34] or decreased levels of infection [30], [34], [35], presumably depending on optimal growth conditions of the invading parasite. For example, experimentally introduced nematodes (*Trichinella spiralis*) rarely develop into adults in bats held at 26°C or below. However, although low temperatures are inhibitory, they are not lethal to the parasite [36]. It is possible that parasites with similar temperature requirements may be able to resume normal development when a torpid host becomes euthermic, either during interbout arousals or at emergence from hibernation in spring. It is also possible that pathogens with optimal growth requirements matching conditions in a torpid bat could thrive by evading host immune responses if bats, like other mammalian hibernators, experience reduced immunity during torpor.

**Figure 1:**
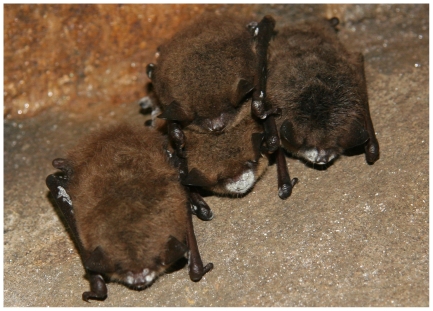
Little brown myotis affected by WNS. Hibernating little brown myotis (*Myotis lucifugus*) with and without visible white fungal growth characteristic of white-nose syndrome.

We evaluated the innate immune responses of hibernating *M. lucifugus* (little brown myotis; Fig. 1) by measuring the activity of plasma complement proteins using bactericidal assays with *Escherichia coli* and *Staphyloccocus aureus* and a fungicidal assay with *Candida albicans* in mid- to late winter 2008 and throughout the winter of 2008–2009. Using these metrics of relative innate immune function, we tested the following hypotheses related to immunocompetence in bats hibernating in WNS-affected sites: (1) bats hibernating at sites where WNS has not been observed (herein referred to as unaffected sites) have stronger relative immune responses compared with bats at WNS-affected sites, where presence of *G. destructans* and unusual rates of mortality have been confirmed; (2) bats with visible cutaneous fungal infections characteristic of WNS (herein referred to as symptomatic bats) have weaker relative immune responses compared with bats without (herein referred to as asymptomatic bats); (3) the ability to mount an immune response is positively correlated with relative body condition; and (4) hibernating bats are less capable of mounting innate immune responses against fungi compared with gram-negative or gram-positive bacteria. To test these hypotheses, we measured bactericidal and fungicidal abilities of blood plasma from *M. lucifugus* collected at affected sites compared with blood plasma from *M. lucifugus* collected at unaffected sites. We also compared symptomatic with asymptomatic bats within affected sites and assessed bactericidal and fungicidal abilities across the hibernation period.

## Materials and Methods

### Field Sample Collection: Winter 2007–2008

Protocols used for capturing, handling and sample collection were reviewed and approved by the Boston University Institutional Animal Care and Use Committee (IACUC approval # 08-022) and followed the United States Fish and Wildlife Service Disinfection Protocol for Bat Studies. Male and female *M. lucifugus* were collected from known affected mines during late hibernation 2008: William's Hotel, Ulster County, New York on February 12, 2008 (*n* = 6); William's Lake, Ulster County, New York on February 13, 2008 (*n* = 28); William's Preserve, Ulster County, New York on February 14, 2008 (*n* = 18); and, Chester Mine, Hampden County, Massachusetts on March 26, 2008 (*n* = 12). Bats were collected from the unaffected Ely Copper Mine, Orange County, Vermont on February 26, 2008 (*n* = 21) and Barton Hill Mine, Albany County, New York on March 25, 2008 (*n* = 18).

All bats were collected by hand from roost substrates and sacrificed by decapitation. Carcasses were stored at −20°C and used for other aspects of our research, including body composition and genetic analyses. Blood samples were collected into heparinized microcapillary tubes (70 µl capacity), centrifuged for three minutes using a portable centrifuge (LW Zipocrit, Lawrenceville, GA) to separate plasma from formed elements, and frozen at −20°C until assayed for complement protein activity. In six bats from Ely Copper Mine, 12 bats from Chester Mine and six bats from William's Preserve Mine, arousal from torpor was stimulated using chemical heating pads for another aspect of our research. Body surface temperature was measured using a thermal infrared camera (Thermacam S-60; FLIR Systems, Inc., Billerica, MA) and bats were sacrificed at 10 minute intervals starting at 0 minutes and ending at 50 minutes from initial collection (across all sites, four bats were sampled at each of the different times). For each bat, body mass (g), length of forearm (mm), sex, and hematocrit were recorded. Each bat was also visually examined for signs of the fungal infection characteristic of WNS, apparent as white, powdery or filamentous hyphae on the nose, ears, wings, or tail membrane. Individuals that exhibited obvious signs of fungal growth were defined as symptomatic. Individuals that did not exhibit such signs were considered asymptomatic for the purposes of our comparisons.

### Field Sample Collection: Winter 2008–2009

Adult female *M. lucifugus* were collected from the following affected sites during the winter of 2008–2009 (See Table 1): William's Hole Six Mine, Ulster County, New York on December 17, 2008 (*n* = 18); Aeolus Cave, Bennington County, Vermont on 18 November, 2008, 31 January, 2009, and 27 March, 2009 (*n* = 58); Chester Mine, Hampden County, Massachusetts on 20 November, 2008 and 2 February, 2009 (*n* = 37); Hibernia Mine, Morris County, New Jersey on 13 January, 2009, and 16 March, 2009 (*n* = 38). Adult female *M. lucifugus* were collected from the unaffected CS&M Mine, Lawrence County, Pennsylvania on 21 January 2009 and 17 March 2009 (*n* = 36) and the unaffected Jones Adit/Vulcan Mine, Dickinson County, Michigan on 25 January 2009 (*n* = 19). For the purposes of this study we considered November and December as early hibernation, January and February as mid-hibernation, and March as late hibernation.

**Table 1:** Sample sizes for winter 2008–2009 collections across multiple hibernacula and different stages within the hibernation period.

Hibernacula	Location	WNS Status	Hibernation Stage	Date	Total Sample Size	Symptomatic Sample Size	Asymptomatic Sample Size
Aeolus Cave	Dorset County, Vermont	affected	early	18-Nov	18	0	18
			mid	31-Jan	21	15	6
			late	27-Mar	19	15	4
William's Six Mine	Ulster County, New York	affected	early	17-Dec	18	6	12
			mid		0	0	0
			late		0	0	0
Chester Mine	Hampden County, Massachusetts	affected	early	20-Nov	18	1	16
			mid	2-Feb	19	9	10
			late		0	0	0
Hibernia Mine	Morris County, New Jersey	affected	early		0	0	0
			mid	13-Jan	19	2	16
			late	16-Mar	19	16	2
CS&M Mine	Lawrence County, Pennsylvania	unaffected	early		0	na	na
			mid	21-Jan	18		
			late	17-Mar	18		
Jones Adit/Vulcan Mines	Dickinson County, Michigan	unaffected	early		0	na	na
			mid	25-Jan	19		
			late		0		

Bats were collected by hand from roost substrates and individually placed in cloth bags. On each sampling date, 18 adult female *M. lucifugus* were sampled, six each corresponding to the following rectal temperatures: 1) below 10°C, 2) 10–20°C and 3), above 20°C to test the hypothesis that body (rectal) temperature affected immune responses for another aspect of our research. Rectal temperature of individual bats at the time of sacrifice was included in statistical analyses to control for the effect of this variable, or was determined to be insignificant before running nonparametric tests. Arousal from torpor was stimulated using an electric heating pad and insulated bag. Rectal temperature was measured using a small thermocouple (Omega HH82A, Stamford, CT) before and after individuals were sacrificed by decapitation. When bats reached the desired rectal temperature they were sacrificed immediately, except in the case of bats sampled near 37°C, which were held for no longer than 30 minutes before sacrifice. General life-history data were collected, including body mass (g) and length of forearm (mm) and all bats were visually inspected for the fungal growth and wing damage characteristic of WNS [9]. Bats from affected sites were categorized as symptomatic or asymptomatic as described above. Blood samples were centrifuged for 3 minutes to separate plasma from formed elements and hematocrit was recorded. Plasma and red blood cell fractions were separated and frozen on dry ice or in a dry-shipper liquid nitrogen dewer during field collection and transportation, then stored at −80°C until assays were performed. Carcasses were stored at −80°C and used for body composition and genetic analyses and the remainder of stored blood was used to assess other aspects of immune function.

### Measuring Bactericidal and Fungicidal Ability

Circulating complement protein activity of plasma was tested using bactericidal and fungicidal competence assays [37] with *Escherichia coli* (Microbiologics product # 0483E7; ATCC # 8739), *Staphylococcus aureus* (Microbiologics product # 0485 E7; ATCC # 6538), or *Candida albicans* (Microbiologics product # 0443 E7; ATCC # 10231). Because samples were frozen prior to assay, phagocytic activity of immune function cells were not responsible for killing target microbes using this method, and a validation using heat-inactivated blood plasma from *Eptesicus fuscus* demonstrated that little to no killing occurs as a result of the presence of heat-stable proteins (M.S. Moore unpublished data). We therefore considered this assay a measure specifically of complement protein activity. All samples were assayed within 33 days of collection, except samples collected from Aeolus Cave, William's Six Mine and Chester Mine during early hibernation that exhibited evidence of contamination upon initial assay and were assayed a second time within 109 days from sample collection. Sterile technique was used for all steps in the protocol. Individual microbes were diluted so that 200–300 colony-forming units were present in 50 µl of solution and blood plasma was diluted 1∶100 using 3 µl plasma in 297 µl 4 mM L-glutamine (Sigma product # G7513) enriched CO_2_-independent media (Gibco product # 18045-088). Bacterial or fungal dilutions and plasma dilutions were mixed 1∶15 using 20 µl of microbial dilution and 280 µl of plasma dilution. Immediately after mixing, one plate for each sample was prepared using a 50 µl aliquot of plasma/microbe mixture spread on tryptic-soy (*E. coli*, *S. aureus*) or Sabboraud-dextrose (*C. albicans*) agar. After the plasma/microbe mixtures were incubated for 60 minutes at 23–25°C, a second set of plates was prepared in triplicate for each sample. Control solutions were prepared using 70 µl microbial dilution and 980 µl media. Using 50 µl aliquots, six control plates were prepared immediately after dilution and six plates were prepared after the 60-minute incubation period. All plates were incubated at 23–25°C for ∼24 hours for *E. coli*, ∼48 hours for *S. aureus*, and ∼72 hours for *C. albicans*. The number of colony-forming units on each plate was recorded visually, means of 60-minute experimental plate sets and 0-minute and 60-minute control plate sets were calculated, and the bactericidal or fungicidal ability of blood plasma was determined as follows: bactericidal/fungicidal ability
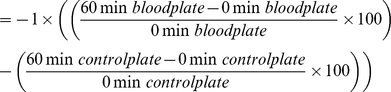
The activity of complement proteins within plasma can kill microbes or inhibit microbial growth resulting in zero or positive killing values. Killing values can also be negative, reflecting an inability of plasma components to kill or inhibit microbes as well as potential microbial division.

### Statistical Analyses

All statistical analyses were conducted using SPSS version 16.0.2 (2008; IBM, Armonk, New York) and graphs were produced using JMP 5.0 (2002; SAS, Carey, North Carolina). For late winter 2007–2008 data, independent samples t-tests were used to test for differences in bactericidal ability against *E. coli* between bats collected from affected and unaffected sites and to compare symptomatic with asymptomatic bats within affected sites (William's Lake and William's Preserve only).

For winter 2008–2009 data, bactericidal ability against *E. coli* and *S. aureus* were normally distributed (Shapiro-Wilk's *p*>0.25), but fungicidal ability against *C. albicans* was not (Shapiro-Wilk's *p* = 0.023). Transformations did not improve distribution of the *C. albicans* data set, and therefore we used nonparametric statistics. For the *E. coli* and *S. aureus* data sets, we used a parametric mixed model ANOVA/ANCOVA, testing the following variables for significance: site, perpetual date, hibernation stage, body temperature, body mass index (BMI; mass (g)/length of forearm (mm)), number of days between blood sample collection and performing assays (DTA; 15–109 days for *E. coli* assays, 16–104 days for *S. aureus* assays, and 20–33 days for *C. albicans* assays), hematocrit, sites categorized as affected vs. unaffected, and individuals categorized as symptomatic vs. asymptomatic (from affected sites only). We also tested for interactions and used a nested design to test for differences between affected and unaffected sites while controlling for between site differences. We used Type IV sums of squares to account for uneven sample sizes between groups and an unbalanced design with missing treatments (e.g. no bats were sampled during early hibernation from unaffected sites). Comparisons of interest were extracted using simple contrasts and each contrast was examined for significance. If Levene's test indicated a significant departure from equal variance (*p*<0.05), we used Brown-Forsythe tests [38] to confirm significance. To further determine differences and relationships between immune response and variables included in reduced models, the Tukey method of post hoc analysis was used. Dependent samples t-tests were used to test for differences in killing ability of blood against the three microbes. These comparisons were performed on all available samples that were tested for killing ability against multiple microbes regardless of hibernation stage, temperature, site and presence or absence of WNS symptoms. Mean percent and standard errors or 95% confidence intervals are presented.

## Results

### Preliminary Results from Winter 2007–2008: Bactericidal ability against E. coli

The bactericidal ability against *E. coli* of blood plasma from bats collected in late hibernation at the affected William's Lake and William's Preserve sites (18%±4%; *n* = 36) was significantly higher than blood plasma from bats collected also in late hibernation at the unaffected Ely Copper Mine (4%±2%; *n* = 21; Mann-Whitney U = 209; *p* = 0.005). When we compared symptomatic and asymptomatic bats from the affected sites only, bactericidal ability of blood plasma from symptomatic bats (10%±4%; *n* = 22) was lower than in asymptomatic bats (25%±6%; *n* = 14; Mann-Whitney U = 97; *p* = 0.062), but this difference was not statistically significant.

### Winter 2008–2009: Bactericidal ability against E. coli and S. aureus and fungicidal ability against C. albicans; Statistical Models for Whole Data Sets

Results for the bactericidal ability of *M. lucifugus* blood plasma against *E. coli* and *S. aureus* were normally distributed. The reduced model, which included hibernation stage, an interaction between hibernation stage and body mass index (BMI), and number of days between sample collection and assay (DTA) significantly explained variation in bactericidal ability of blood plasma against *E. coli* across all samples (*n* = 177; F*_10, 170_* = 13.45; *p*<0.001; R^2^ = 0.32; adjusted R^2^ = 0.30). The reduced model including site, hibernation stage and DTA significantly explained variation in bactericidal ability of *M. lucifugus* blood plasma against *S. aureus* across all samples (*n* = 171; F*_8, 151_* = 29.03; *p*<0.001; R^2^ = 0.59; adjusted R^2^ = 0.57). Results for the fungicidal ability of *M. lucifugus* blood plasma against *C. albicans* were non-normally distributed. Thus, nonparametric statistics were used to analyze this data set (see below). Body temperature was not significantly related to any of the tested microbicidal abilities.

### Differences between WNS-affected and unaffected sites

**Figure 2:**
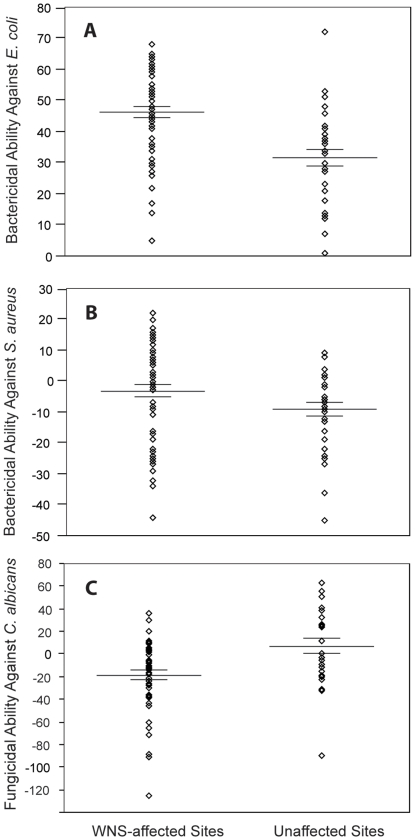
Microbicidal ability of blood plasma from little brown myotis collected in WNS-affected and unaffected sites. Microbicidal ability, expressed as percent microbe killed, against *E. coli* (A), *S. aureus* (B), and *C. albicans* (C) in *M. lucifugus* collected from WNS-affected and unaffected sites during the winter of 2008–2009. Significantly more *E. coli* was killed by plasma collected from bats hibernating in WNS-affected sites compared with plasma collected from bats hibernating in unaffected sites (affected *n* = 54; unaffected *n* = 31; F*_1, 80_* = 16.22; *p*<0.001; R^2^ = 0.17). Bactericidal ability against *S. aureus* was also significantly greater in bats collected from affected sites compared with bats from unaffected sites (affected *n* = 55; unaffected *n* = 31; F*_1, 81_* = 9.05; *p* = 0.004; R^2^ = 0.10), but fungicidal ability against *C. albicans* was significantly lower in bats collected at affected sites compared with bats collected at unaffected sites (affected *n* = 52; unaffected *n* = 25; Mann-Whitney U = 383; *p* = 0.004). Lines indicate mean percent microbe killed ±1 SE.

To test the hypothesis that bactericidal ability against *E. coli* and *S. aureus* differs between bats from WNS-affected and unaffected sites, we used samples only from mid- hibernation because: (1) hibernation stage was a significant predictor in the reduced models, (2) high mortality or logistical challenges prevented collection of samples from all sites during each hibernation stage, and (3) there was a much smaller range in DTA (15–23 for *E. coli* assays and 16–31 for *S. aureus* assays) during mid-hibernation. Within the mid-hibernation subset, we found that bactericidal ability of blood plasma against *E. coli* was significantly greater in bats from WNS-affected sites compared with bats from unaffected sites (affected: 45%±2%, *n* = 54; unaffected: 31%±3%, *n* = 31; F*_1, 80_* = 16.22; *p*<0.001; R^2^ = 0.17; Fig. 2a) after controlling for the effect of DTA and site. No significant difference in bactericidal ability against *E. coli* was observed among individual sites (F*_3, 80_* = 1.55; *p*<0.207; R^2^ = 0.06). Bactericidal ability of blood plasma against *S. aureus* was also significantly greater in bats collected from affected sites compared with those from unaffected sites (affected: −3%±2%, *n* = 55; unaffected: −9%±2%, *n* = 31; F*_1, 81_* = 9.05; *p* = 0.004; R^2^ = 0.10; Fig. 2b) after controlling for the effect of DTA and site. However, within this data set, heterogeneity among individual sites explained more of the variation in bactericidal ability than did site type (F*_3, 81_* = 27.68; *p*<0.001; R^2^ = 0.51). We did not detect significant variation in fungicidal ability against *C. albicans* across the hibernation period (see below), and therefore used all available samples (mid- and late hibernation; DTA range = 20–33) to test for differences between site types. Fungicidal ability against *C. albicans* was significantly lower in blood plasma from bats collected at affected sites compared with blood plasma from bats collected at unaffected sites (affected *n* = 52, −19%±4%; unaffected *n* = 25, 7%±7%; Mann-Whitney U = 383; *p* = 0.004; Fig. 2c). We also observed significant variation in fungicidal ability of blood plasma against *C. albicans* among individual sites regardless of their WNS status (Kruskal-Wallis χ = 17.15; *p* = 0.001).

### Comparisons between visibly symptomatic and asymptomatic bats

The availability of visibly symptomatic and asymptomatic bats within affected sites varied across the hibernation period, with more asymptomatic bats being available during early hibernation and more symptomatic bats available during late hibernation (See Table 1). Only during mid-hibernation were we able to collect a roughly equal number of each and thus we used this period to test for differences between bats with and without visible symptoms of WNS. When we compared symptomatic with asymptomatic bats within affected hibernacula we found no difference in the bactericidal ability of blood plasma against *E. coli* (symptomatic: 42%±4%, *n = *23; asymptomatic: 48%±3%, *n* = 30; F*_1, 49_* = 1.87; *p*<0.178; R^2^ = 0.04). We also found no difference in bactericidal ability of blood plasma against *S. aureus* between symptomatic and asymptomatic bats (symptomatic: −10%±4%, *n = *24; asymptomatic: 2%±2%, *n = *30; F*_1, 50_* = 0.37; p = 0.547; R^2^ = 0.01). Fungicidal ability of blood against *C. albicans* also did not differ between symptomatic and asymptomatic bats (symptomatic: −18%±5%, *n* = 30; asymptomatic: −17%±8%, *n* = 20; Mann-Whitney U = 287; *p* = 0.80).

### Variation associated with hibernation stage and body condition

**Figure 3:**
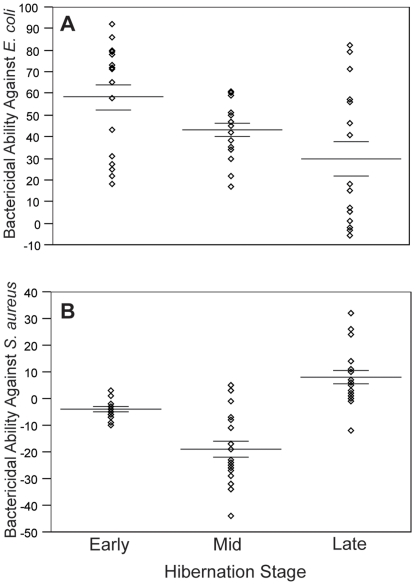
Microbicidal ability across the hibernation period. Microbicidal ability, expressed as percent microbe killed, against *E. coli* (A) and *S. aureus* (B) in blood plasma collected from little brown myotis hibernating in the affected Aeolus Cave during the 2008–2009 hibernation period. Bactericidal ability of blood plasma against *E. coli* varied significantly throughout the hibernation period (early *n* = 18; mid *n* = 18; late *n* = 16; F*_2, 49_* = 5.86; *p* = 0.005; R^2^ = 0.19) and was greater during early hibernation than in late hibernation (*p* = 0.004). Bactericidal ability of blood plasma against *S. aureus* also varied significantly throughout the hibernation period (early *n* = 18; mid *n* = 18; late *n* = 17; F*_2, 51_* = 31.7; *p*<0.001; R^2^ = 0.55), but was greater in early hibernation compared with mid-hibernation (*p*<0.001) and greater in late hibernation than in early hibernation (*p* = 0.002) and mid-hibernation (*p*<0.001). Lines indicate mean percent microbe killed ±1 SE.

We were only able to acquire samples across the entire hibernation period from the affected Aeolus Cave and therefore used this site to test for variation related to hibernation stage (early, mid, and late). Bactericidal ability of blood plasma against *E. coli* varied throughout the hibernation period (early: 58%±6%, *n* = 18; mid: 43%±3%, *n* = 17; late: 30%±8%, *n* = 16; F*_2, 48_* = 6.14; *p* = 0.004; R^2^ = 0.20; Fig. 3a) and was greater during early hibernation than in late hibernation (*p* = 0.004). Bactericidal ability of blood plasma against *S. aureus* also varied significantly throughout the hibernation period (early: −4%±1%, *n* = 18; mid: −19%±3%, *n* = 18; late: 8%±3%, *n* = 17; F*_2, 51_* = 31.7; *p*<0.001; R^2^ = 0.55; Fig. 3b) and was greater in early hibernation compared with mid-hibernation (*p*<0.001), but greater in late hibernation than in early hibernation (*p* = 0.002) and mid-hibernation (*p*<0.001). We did not test blood plasma collected during early hibernation for fungicidal ability against *C. albicans* and, for this reason, did not evaluate variation in this response across the entire hibernation period. However, fungicidal ability of blood plasma against *C. albicans* did not differ between mid- (*n* = 26; −9%±4%) and late hibernation (*n* = 51; −11%±6%; Mann-Whitney U = 628; *p* = 0.706).

Because an interaction variable including BMI and hibernation stage was a significant predictor in the reduced model for the entire *E. coli* data set (F*_3, 166_* = 3^.^664; *p* = 0.014; R^2^ = 0.062), we investigated patterns in BMI across sites and across the hibernation period. We then tested the relationship between bactericidal ability of blood plasma against *E. coli* and BMI within each hibernation stage. BMI was significantly higher in unaffected bats compared with affected bats during mid- (affected *n* = 59, 0.18±0.01; unaffected *n* = 35, 0.22±0.03; F*_1, 93_* = 79.19; *p*<0.001) and late hibernation (affected *n* = 38, 0.18±0.01; unaffected *n* = 18, 0.19±0.01; F*_1, 55_* = 29.13; *p*<0.001). Controlling for site, BMI varied throughout the hibernation period in bats from affected sites (early *n* = 42, 0.21±0.02; mid *n* = 59, 0.18±0.01; late *n* = 38, 0.18±0.01; F*_7, 140_* = 25.52; *p*<0.001; R^2^ = 0.58). Specifically, BMI was significantly higher in affected bats collected during early hibernation compared with mid- and late hibernation (*p*<0.001), but did not differ between affected bats collected in mid- and late hibernation (*p* = 0.344). After controlling for site, BMI did not vary across hibernation stages in bats collected from unaffected sites (mid: 0.21±0.03, *n* = 35; late: 0.19±0.01, *n* = 18; *p* = 0.211).

**Figure 4:**
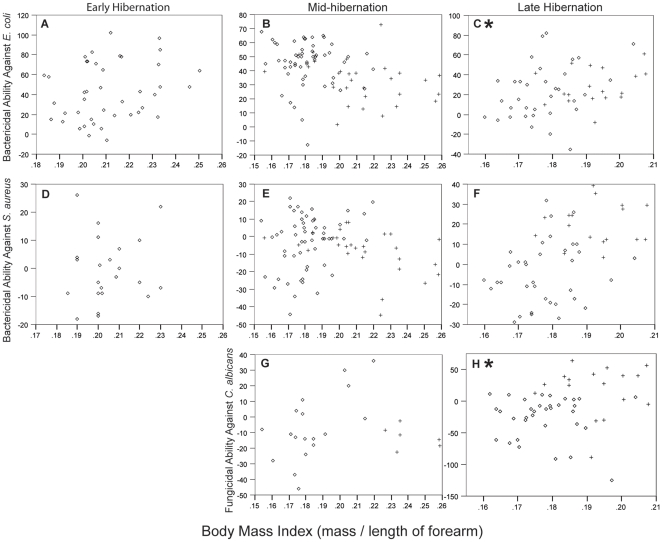
Microbicidal ability and body condition. Microbicidal ability, expressed as percent microbe killed, against *E. coli* (A–C), *S. aureus* (D–F), and *C. albicans* (G, H) in little brown myotis plotted against BMI. After controlling for site, BMI was not significantly related to bactericidal ability against *E. coli* in early hibernation (F*_1, 38_* = 0.12; *p* = 0.745) or mid-hibernation samples (F*_1, 77_* = 2.87; *p* = 0.094) but was positively related to BMI in late hibernation samples (F*_1, 48_* = 4.47; *p* = 0.04; R^2^ = 0.085). After controlling for site, BMI did not correlate with bactericidal ability against *S. aureus* during any stage of hibernation. Fungicidal ability of blood against *C. albicans* was not associated with BMI during mid-hibernation, (*n* = 24; Spearman's ρ = 0.14, *p* = 0.527), but was significantly and positively associated with BMI during late hibernation (*n* = 51; Spearman's ρ = 0.29, *p* = 0.039). Open diamonds indicate samples collected from bats hibernating in WNS-affected sites and crosses indicate samples collected from bats hibernating in unaffected sites.

After controlling for site, BMI was not significantly related to bactericidal ability against *E. coli* in early hibernation (F*_1, 38_* = 0.12; *p* = 0.745) or mid-hibernation samples (F*_1, 77_* = 2.87; *p* = 0.094) but was positively related to BMI in late hibernation samples (F*_1, 48_* = 4.47; *p* = 0.04; R^2^ = 0.09; Fig. 4a–c). When we tested this relationship in bats from affected and unaffected sites separately, we observed a positive, although non-significant, trend in bactericidal ability of blood against *E. coli* during late hibernation from bats collected at affected sites (*n* = 34; F*_1, 31_* = 3.24; *p* = 0.082; R^2^ = 0.10) but not from bats collected at unaffected sites (*n* = 18; F*_1, 16_* = 1.19; *p* = 0.291; R^2^ = 0.07). After controlling for site, BMI did not correlate with bactericidal ability against *S. aureus* during any stage of hibernation (Fig. 4d–e). Fungicidal ability of blood against *C. albicans* was not associated with BMI during mid-hibernation, (*n* = 24; Spearman's ρ = 0.14, *p* = 0.527), but was positively associated with BMI during late hibernation (*n* = 51; Spearman's ρ = 0.29, *p* = 0.039; Fig. 4f,g). In bats from affected hibernacula, fungicidal ability of blood against *C. albicans* was positively correlated with BMI during mid-hibernation (*n* = 18; Spearman's ρ = 0.47, *p* = 0.048), but not during late hibernation (*n* = 34; Spearman's ρ = 0.04, *p* = 0.808). Blood plasma from bats collected at unaffected sites showed no association between BMI and fungicidal ability against *C. albicans* during mid-hibernation (*n* = 6; Spearman's ρ = −0.20, *p* = 0.70) or during late hibernation (*n* = 17; Spearman's ρ = 0.11; *p* = 0.68). However, because we were unable to transform *C. albicans* data to achieve a normal distribution, we were unable to test if these relationships were significant after controlling for site.

### Response to different microbes

**Figure 5:**
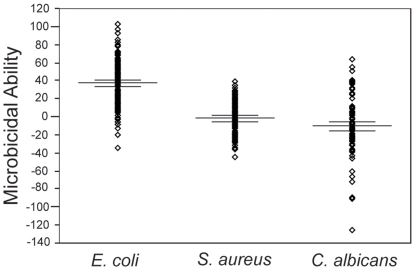
Microbicidal ability against different microbe types. Microbicidal ability against the three different microbes *E. coli*, *S. aureus* and *C. albicans* of blood plasma collected from hibernating little brown myotis across all sites and stages of hibernation. Plasma from *M. lucifugus* was significantly better at killing *E. coli* compared to *S. aureus* (*n* = 168; *t* = 18.22; df = 167; *p*<0.001), significantly better at killing *E. coli* compared to *C. albicans* (*n* = 75; Wilcoxon *Z* = −6.59; *p*<0.001), and significantly better at killing *S. aureus* compared to *C. albicans* (*n* = 75; Wilcoxon *Z* = −2.94; *p* = 0.003). Lines indicate mean percent microbe killed and 95% confidence intervals.

**Figure 6:**
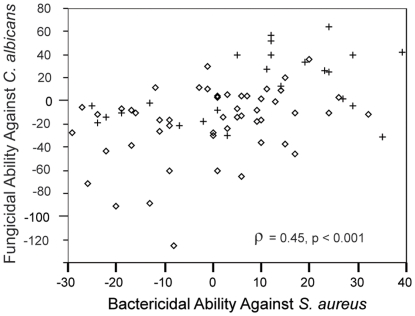
Microbicidal ability against a gram-positive bacteria and a fungus. Correlation between bactericidal ability of plasma against *S. aureus* and fungicidal ability of plasma against *C. albicans*, both expressed as percent microbe killed (Spearman's ρ = 0.45, p<0.001). Open diamonds represent samples collected from little brown myotis hibernating in WNS-affected sites and crosses represent samples collected from little brown myotis hibernating in unaffected sites.

Mean killing ability of blood plasma from hibernating bats across all sites and stages of hibernation against the three different microbes used in this study were as follows: *E. coli*: 37%±2%; *S. aureus*: −2%±1%; *C. albicans*: −10%±4%. Blood plasma from *M. lucifugus* was significantly better at killing *E. coli* compared to *S. aureus* (*n* = 168; *t* = 18.22; df = 167; *p*<0.001), significantly better at killing *E. coli* compared to *C. albicans* (*n* = 75; Wilcoxon *Z* = −6.59; *p*<0.001), and significantly better at killing *S. aureus* compared to *C. albicans* (*n* = 75; Wilcoxon *Z* = −2.94; *p* = 0.003; Fig. 5). There was a significant positive correlation between bactericidal ability of blood plasma against *S. aureus* and fungicidal ability against *C. albicans* (Spearman's ρ = 0.454, p<0.001; Fig. 6).

## Discussion

Alterations in innate immune function, as measured by complement protein activities against gram-negative bacteria (*E. coli*), gram-positive bacteria (*S. aureus*), and fungi (*C. albicans*) appear to be associated with the development of WNS in hibernating *M. lucifugus*. On average, blood plasma from *M. lucifugus* hibernating in WNS-affected sites had significantly greater ability to activate complement proteins against *E. coli* compared with blood plasma from bats hibernating in unaffected sites. Blood plasma from bats hibernating in WNS-affected sites also had significantly greater ability to kill *S. aureus* through complement protein activity compared with blood plasma from bats collected at unaffected sites, although differences between sites where bats were collected accounted for more of the variation in bactericidal ability against *S. aureus* than hibernacula type (WNS-affected vs. unaffected). In contrast to differences in bactericidal abilities, blood plasma from bats collected from WNS-affected sites was significantly less able to kill the fungus *C. albicans* compared with blood plasma from bats collected at unaffected sites; however, in this case we were unable to control for site while testing this relationship because of the non-normal distribution of the *C. albicans* data set. The observed differences in microbicidal abilities between bats hibernating at affected and unaffected sites (specifically during mid-hibernation) may be explained in at least two ways. First, it is possible that *G. destructans* is eliciting an immune response that involves increases in complement protein production and activity in blood. Increases in complement protein production and activity in blood may be particular only for the responses that are also used against *E. coli* and *S. aureus*, or all aspects of complement may be upregulated and there is some disruption in the activity associated with lysing *C. albicans* resulting in the relative decrease in this measure. It is also possible that blood proteins involved in killing *C. albicans* were increased but already activated against *G. destructans* and therefore incapable of activating against and lysing *C. albicans*. Alternatively, given that preliminary evidence shows that bats hibernating in affected sites arouse from torpor more frequently compared to unaffected bats [1], greater use of euthermia may result in increased circulation of complement proteins [24]. If this were the sole explanation for altered complement protein activity, it seems likely that in our experiments we would have observed elevated fungicidal ability against *C. albicans* as well. Still, it is possible that increased use of euthermia resulted in relatively higher levels of complement proteins in circulation while a disruption or previous activation reduced the availability of certain complement proteins in blood to attack *C. albicans*. Although revealing significant variation in responses against different microbes between hibernacula types, unfortunately this field-based evidence does not allow us to differentiate between the possible explanations presented here. Accordingly, we stress that controlled laboratory-based methods are necessary to determine exactly what alterations are occurring in response to *G. destructans* and how potential changes in torpor patterns contribute to variation in complement protein activity in the blood of hibernating *M. lucifugus*.

Across all microbes used to assess complement protein activity of blood plasma, there was no difference in microbicidal abilities between visibly symptomatic compared to asymptomatic bats within affected sites. One hypothesis for this observation is that there is no underlying difference in immune function between the groups and that bats without visible fungal infections may have been more recently aroused from torpor at which time they groomed superficial fungus from their skin. We do not know the recent history of torpor in the free-ranging animals that we sampled to evaluate whether this is a valid explanation. Additionally, there may be differences among individuals regarding development of fungal infections that relate to differences in immune responses aside from complement protein activity or that occur during different stages within the hibernation period. However, because of the disparity in sample sizes between the two groups within early and late hibernation, we could only test for differences between these two groups during mid-hibernation. Again, by monitoring and controlling for variation in use of torpor, laboratory-based experiments may elucidate potential differences between individuals in immune parameters that facilitate or inhibit fungal invasion.

We observed a significant decrease in bactericidal ability of blood against *E. coli* as the hibernation season progressed in samples collected from the affected Aeolus Cave. Bactericidal ability of blood plasma against *S. aureus* also varied throughout the course of hibernation in bats from Aeolus Cave, but was lowest during mid-hibernation and highest in late hibernation. We did not test blood plasma for variation in fungicidal ability against *C. albicans* across the hibernation period because we were modifying the killing assay for use with this microbe during early hibernation, and because we were unable to collect blood from unaffected bats during early hibernation, we did not test for variation across the hibernation period in this group. In the case of bactericidal ability against *E. coli*, if bats increased production and activity of complement proteins in response to *G. destructans* or another pathogen, this response may have occurred prior to or during early hibernation and contributed to the observed higher responses in bactericidal ability against *E. coli* during early hibernation. Increased complement protein production and activity in blood plasma from affected bats may have also contributed to depleted energy reserves and decreased their capability of maintaining higher bactericidal abilities against *E. coli* into mid- and late hibernation. Alternatively, it is possible that complement proteins in blood plasma decrease naturally across the hibernation period because of the extensive use of torpor and overall reduction in protein synthesis [39]; however, we were unable to test this hypothesis in unaffected bats due to the rapid spread of the syndrome during the winter of 2008–2009. Patterns in bactericidal ability against *S. aureus* are more challenging to interpret in light of either hypothesis given the nonlinear change in bactericidal ability of blood plasma across this hibernation period; however, the range of proportion bacteria killed was much smaller for *S. aureus* (between −44% and 32%) compared with *E. coli* (between −13% and 92%) and bactericidal abilities of blood plasma against *S. aureus* were more often negative compared with bactericidal abilities against *E. coli*. Both of these occurrences may have reduced our ability to detect biologically meaningful variation across the hibernation period when using killing assays with *S. aureus*. In future studies, the use of more concentrated plasma samples in *S. aureus* killing assays may increase variation and help elucidate potential differences between groups.

This study also demonstrated significant correlations between complement protein activity of blood plasma against *E. coli* and *C. albicans* and body mass index (BMI) in hibernating *M. lucifugus*, but no significant relationships between bactericidal ability against *S. aureus* and BMI in the same bats. In tests of the bactericidal ability of blood plasma against *E. coli*, where we were able to control for the differences among sites, we found a positive correlation with BMI during late hibernation. We also observed a positive correlation between fungicidal ability of blood plasma against *C. albicans* and BMI during late hibernation, but were unable to control for differences among sites using this method. Little is known about the energetic costs of immunity or how the immune system interacts with and depends on metabolic rate and energy availability [40], [41]; however, studies on a number of vertebrate and invertebrate taxa have demonstrated increased metabolic rate [42], [43], [44], [45], [46] and decreased body mass [44], [46], [47] during immunological stimulation. Ots *et al*. [46] demonstrated that immune challenge increased basal metabolic rate (BMR) in wintering great tits (*Parus major*) injected with the novel antigen sheep red blood cells (SRBC) and that larger responses against the antigen, measured as antibody titers against SRBC and changes in heterophil to lymphocyte ratios, were associated with greater loss of body mass. Similar increases in BMR have also been observed in collared doves (*Streptopelia decaocto*) injected with SRBC [46], mice injected with keyhole limpet hemocyanin (KLH) [42], and house sparrows (*Passer domesticus*) injected with phytohemagglutinin (PHA) [45]. Bonneaud *et al*. [47] demonstrated significant body mass loss related to immune response in house sparrows (*Passer domesticus*) injected with lipopolysaccharide derived from *E. coli*. Several studies have also shown lower immune response in animals with reduced fat reserves or reduced energy availability [48], [49], [50]. Demas *et al*. [49] demonstrated that experimental reductions in total body fat reduced the ability of prairie voles (*Microtus ochrogaster*) and Siberian hamsters (*Phodopus sundorus*) to mount humoral immune responses. Measuring response to PHA injections in Siberian gerbils (*Meriones unguiculatus*), Xu and Wang [50] found that fasting gerbils produced significantly smaller swellings in comparison to fed gerbils, and that response to PHA was positively correlated with amount of body fat. Zysling and Demas [48] experimentally reduced energy availability using 2-deoxy-D-glucose in long- and short-day Siberian hamsters (*Phodopus sungorus*) injected with KLH and observed lower antibody responses in long-day but not short-day animals. Moreover, in addition to providing free fatty acids to lymphocytes as fuel [51] and cell wall components [52], adipocytes are known to secrete proteins such as leptin, a neuroendocrine signal that indicates current energy reserve levels to the immune system, and TNF-α, which mediates inflammatory and cytotoxic immune responses [40], [53], [54]. Additionally, the adipose tissue is integral in the innate production of T helper cell cytokines [55]. These results in combination support the idea that the immune system is affected by and dependent on the relative body condition of an individual, particularly with respect to energy availability, and that immunological stimulation depletes energy reserves and reduces body condition.

Results from studies in other taxa parallel the correlations we observed between immune response and BMI in hibernating *M. lucifugus* and support the notion that considerable trade-offs may be occurring between immune responses and energetics in bats afflicted with WNS. It is possible that all bats sampled at affected sites entered hibernation with sufficient energy reserves and sufficient or even elevated complement proteins in circulation to maintain a certain level of immunological competence. As the hibernation season progressed, affected bats used fat reserves to periodically arouse from torpor, an energetically expensive process [56] that may have contributed to the decrease in mean BMI from early to late hibernation. To date, it is unknown why many bats hibernating in WNS-affected sites experience reduced BMI. However, preliminary results show that bats hibernating in WNS-affected sites arouse from torpor more frequently compared with bats in unaffected sites [1], which would likely cause premature reductions in energy stores to fuel both arousal and sustained euthermia. One possibility is that *G. destructans* elicits immune responses when bats periodically arouse from torpor, which would help explain the elevated bactericidal abilities against *E. coli* and *S. aureus* that we observed in our study. This would likely result in altered interbout arousal periods [28], [29], elevated metabolic rates, and increased fat mobilization and energy expenditure [42], [43], [44], [45], [46]. Alternatively, *G. destructans* may simply irritate hibernating bats, which may arouse from torpor more frequently to groom the fungus from their skin [1]. In any case, affected bats exhaust reserves of both brown and white adipose tissue [3]. As energy reserves become diminished, bats may trade immunity for survival, which would result in individuals with lower BMI exhibiting reduced immune responses. Once individuals exhaust stored energy reserves and experience reductions in immune responses, *G. destructans* would be able to invade cutaneous tissue while evading host immune responses. Final reductions in energy reserves could result in the inability of bats to arouse from torpor (Jonathan D. Reichard and Marianne S. Moore, unpublished data) and death.

We observed highly significant differences in the ability of complement proteins in blood plasma from *M. lucifugus* to lyse *E. coli*, *S. aureus*, and *C. albicans* across all samples. These results demonstrate that hibernating *M. lucifugus* are less capable of responding to gram-positive bacteria (*S. aureus*) than to gram-negative bacteria (*E. coli*), and are least capable of responding to a fungus (*C. albicans*) through complement activity. It would be interesting to determine if these differences are broadly applicable to the ability of bats to respond to these generalized microbe types, or if specific characteristics of the microbes we used resulted in greater or lesser complement protein activation.

The use of microbicidal assays presented in this study is one of many approaches being used to assess relative immune responses in bats affected by WNS. A multifaceted approach to estimate levels of immunocompetence is important, given the inherently complex nature of the immune system [57] and various interactions between host, pathogen and environment [58]. In this study, using three microbes to activate complement proteins has revealed a variety of relationships that would not be apparent using a single approach. Results generated to date support the hypothesis that bats living in affected sites experience reductions in innate immune responses, particularly against the fungus *C. albicans*; however, we also found elevated bactericidal responses in bats hibernating in affected sites suggesting a more complex interaction between immune function and the development of WNS. Our results also show that bats hibernating in WNS-affected sites experience significant variation in bactericidal ability across the hibernation period and that *M. lucifugus* have differing capabilities of neutralizing pathogens through complement protein activity in general, with particularly weak activities against fungi. Our study supports the hypothesis that the physiology of torpor and the relationship between energetics and immune function may promote the development of WNS and control the ability of *M. lucifugus* and other bat species to resist infection by *G. destructans*.
